# Endoplasmic Reticulum Stress in Cancer Progression: A Comprehensive Review of Its Role and Mechanisms

**DOI:** 10.7150/ijms.120874

**Published:** 2025-10-27

**Authors:** Xin Yu, Wenge Li, Shengrong Sun, Juanjuan Li

**Affiliations:** 1 Department of Breast and Thyroid Surgery, Renmin Hospital of Wuhan University, Wuhan, Hubei, P. R. China.; 2 Department of ‌Oncology, Fuzhou Pulmonary Hospital and Fujian Medical University Clinical Teaching Hospital, Fuzhou, Fujian, P. R. China.

**Keywords:** endoplasmic reticulum stress, cancer, tumor microenvironment, unfolded protein response

## Abstract

Endoplasmic reticulum (ER) stress plays a pivotal role in tumor progression. As research in tumor biology advances, the relationship between ER stress and tumor initiation, development, and immune regulation has increasingly attracted attention. ER stress activates the unfolded protein response (UPR), thereby affecting key processes in tumor cells, including metabolism, proliferation, invasion, metastasis, and drug resistance. Moreover, it modulates tumor immune responses by regulating the functions of immune cells within the tumor microenvironment. This review consolidates the concept of ER stress as a central signaling hub that dictates cell fate and extensively remodels the tumor ecosystem. From a clinical perspective, this understanding provides a strong rationale for therapeutically targeting the UPR, suggesting that combining ER stress modulators with immunotherapy represents a promising strategy to overcome therapeutic resistance and improve patient outcomes.

## 1. Introduction

Endoplasmic reticulum (ER) stress represents a cellular response activated in the face of conditions such as the aggregation of misfolded and unfolded proteins within the ER lumen, as well as disruptions in calcium homeostasis^1^. This triggers the unfolded protein response (UPR), a complex signaling network that aims to restore equilibrium in protein function and protect the cell from potential damage^2^. The UPR is controlled by three essential sensors: inositol-requiring enzyme 1 (IRE1), protein kinase RNA-activated (PERK), and activating transcription factor 6 (ATF6). These sensors activate various pathways responsible for managing protein folding, ER-associated degradation (ERAD), oxidative stress, autophagy, and mitochondrial function, depending on the specific stress conditions encountered^2^-^4^.

Cancer remains one of the primary causes of death globally, with rising rates of occurrence and few effective treatment options available for many individuals^5^. The complexity of cancer biology and the heterogeneity of tumors pose significant challenges for effective treatment^6^. One emerging area of research focuses on the role of ER stress in cancer progression^7^-^9^. Studies indicate that ER stress serves as both a cause and a result of cancer progression and metastasis. Oncogenic mutations and cellular transformation often lead to increased protein synthesis and ER stress^10^-^12^, which can promote tumor cell survival and adaptation to the hostile tumor microenvironment (TME)^7^,^13^. Additionally, ER stress contributes to the immune evasion and immune suppression observed in many cancers^7^,^14^-^16^.

This review provides an in-depth exploration of the diverse roles that ER stress plays in cancer development and its therapeutic implications. We will examine the physiological mechanisms underlying ER stress, emphasizing the signaling pathways and cellular processes involved. The discussion will then shift to the effects of ER stress on the TME, with a focus on its influence on tumor cells and immune cells. By understanding the complex interplay between ER stress and cancer progression, we can identify novel therapeutic strategies that exploit this vulnerability to improve patient outcomes.

## 2. Signaling pathways of ER stress

**Figure 1:**
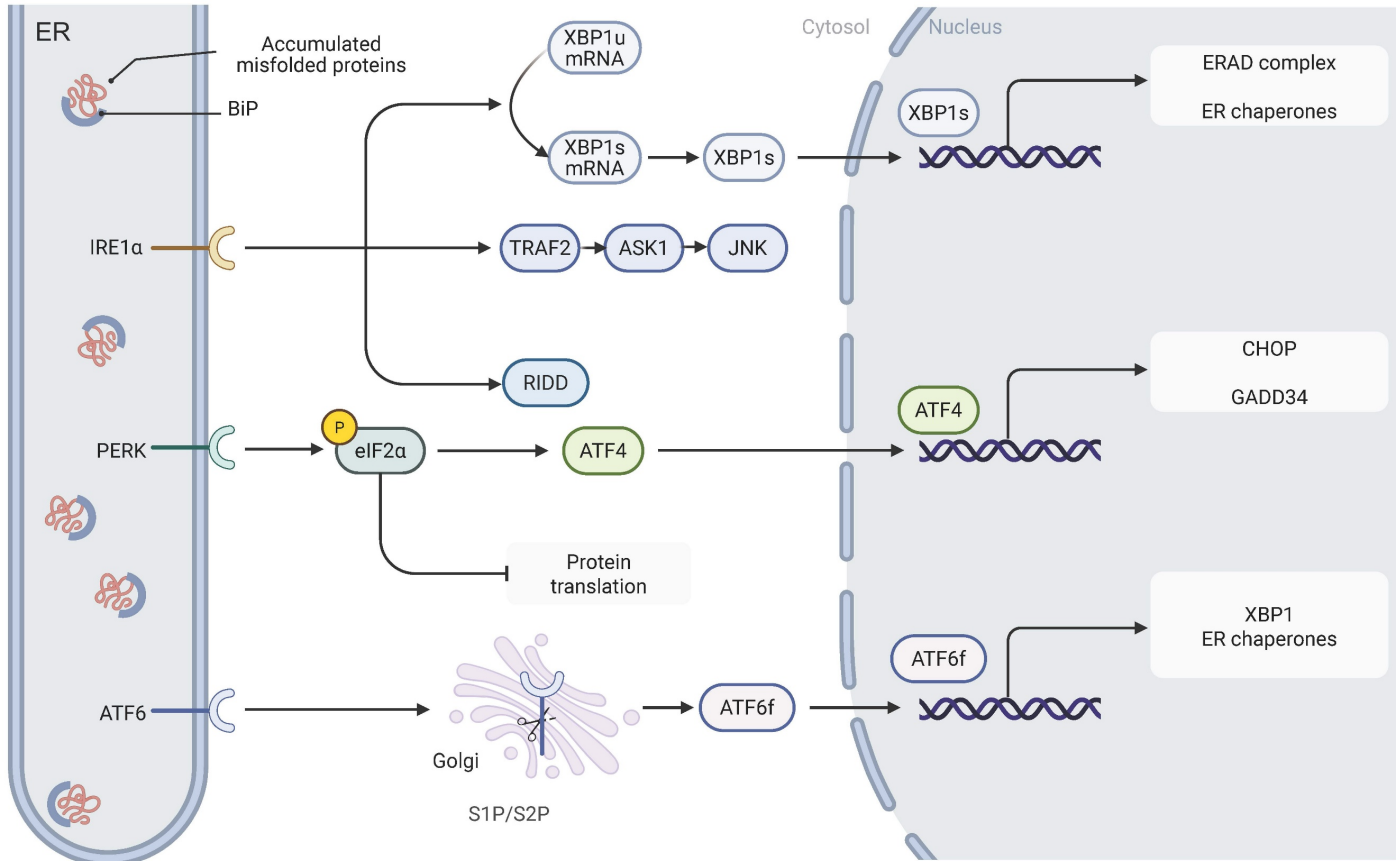
** Signaling pathways of ER stress.** The three key ER stress sensors, IRE1, PERK, and ATF6, are activated upon the accumulation of misfolded proteins in the ER lumen. Upon activation, IRE1 splices XBP1 mRNA, resulting in the formation of the XBP1s transcription factor, which upregulates genes involved in protein folding and ERAD. Additionally, IRE1 activates the JNK signaling pathway through the recruitment of TRAF2 and ASK1, contributing to apoptosis. PERK phosphorylates eIF2α, leading to a reduction in global protein synthesis while selectively enhancing the translation of ATF4, a transcription factor that activates genes such as CHOP and GADD34 involved in protein synthesis regulation and stress response. ATF6 is processed in the Golgi apparatus and, once cleaved, translocates to the nucleus as ATF6f, where it activates UPR-related genes. Together, these pathways coordinate an adaptive response aimed at restoring ER homeostasis.

The ER, as the core functional unit in eukaryotic cells, plays a pivotal role in the regulation of protein synthesis, maturation, and folding^3^. By mediating post-translational modifications such as disulfide bond formation, the ER ensures that proteins acquire functional three-dimensional conformations—a process precisely controlled by multiple factors including ATP, calcium ions, and the oxidative microenvironment. When unfolded or misfolded proteins accumulate abnormally, the aggregation of nascent polypeptide chains triggers ER stress. In response, cells activate the UPR, an adaptive signaling cascade aimed at restoring proteostasis^2^. The UPR is chiefly regulated by three ER-resident stress sensors: IRE1, PERK, and ATF6 (Fig.1). Under homeostatic conditions, these sensors are bound to the ER chaperone glucose-regulated protein 78 (GRP78), maintaining them in an inactive state; however, when ER stress occurs, the accumulation of misfolded proteins causes GRP78 to dissociate from these sensors, thereby initiating the UPR signaling cascade^2^,^3^,^17^.

### 2.1. IRE1-XBP1

IRE1, an ER transmembrane protein, exhibits dual functionalities as a serine/threonine kinase and a RNase. Upon the onset of ER stress, IRE1 is activated through oligomerization and autophosphorylation^18^. Two functional isoforms of IRE1 have been characterized: IRE1α, which is ubiquitously expressed in eukaryotic cells, and IRE1β, which displays a tissue-specific distribution, predominantly localizing to gastrointestinal and respiratory epithelial cells^19^,^20^. Once activated, IRE1 initiates its RNase activity via phosphorylation of its kinase domain, thereby mediating the specific splicing of X-box-binding protein 1 (XBP1) mRNA; this process converts the unspliced form (XBP1u) into the transcriptionally active spliced isoform (XBP1s)^21^. As a transcriptional regulator, XBP1s significantly upregulates genes involved in protein folding, ERAD, and ER protein translocation, thereby playing a crucial role in restoring ER homeostasis^22^. In addition to facilitating XBP1 splicing, IRE1 recruits TNF receptor-associated factor 2 (TRAF2) and apoptosis signal-regulating kinase 1 (ASK1) to activate the c-Jun N-terminal kinase (JNK) and p38 mitogen-activated protein kinase (MAPK) signaling pathways. This cascade induces apoptosis by promoting B-cell lymphoma-2 (Bcl-2) interacting mediator of cell death (BIM) protein activation and repressing Bcl-2 expression^23^,^24^.

### 2.2. PERK-eIF2α-ATF4-CHOP

PERK, an integral membrane protein of the ER, plays a pivotal role in sensing ER stress. When unfolded proteins accumulate abnormally within the ER lumen and trigger a stress response, the molecular chaperone GRP78 dissociates from the PERK complex, thereby initiating its oligomerization and autophosphorylation activation process^25^. Activated PERK phosphorylates eukaryotic translation initiation factor 2 subunit alpha (eIF2α) at serine 51, markedly inhibiting its activity and leading to an overall reduction in protein synthesis. This translational repression alleviates the burden of nascent polypeptides, effectively mitigating the protein processing load on the ER^26^.

Notably, while eIF2α phosphorylation globally suppresses protein synthesis, it selectively enhances the translation of specific mRNAs. For example, ATF4 mRNA contains canonical upstream open reading frames (uORFs) that typically inhibit initiation at the main open reading frame^27^. Upon phosphorylation of eIF2α, the uORF-mediated repression is significantly relieved, thereby promoting efficient translation of ATF4's primary coding region and subsequent protein expression^28^. As a key transcriptional regulator, ATF4 activates various stress response-related genes—including CCAAT/enhancer-binding protein (C/EBP) homologous protein (CHOP) and growth arrest and DNA damage-inducible 34 (GADD34)—whose gene products collectively contribute to the regulation of protein synthesis and the ER stress response^29^.

### 2.3. ATF6

ATF6 is a single-pass transmembrane protein that is constitutively expressed in cells^30^. Under ER stress conditions, ATF6 dissociates from the molecular chaperone GRP78 and is subsequently transported to the Golgi apparatus for proteolytic processing^31^. This cleavage event generates a cytosolic fragment (ATF6 fragment, ATF6f) that contains a transcriptional activation domain (TAD), a basic leucine zipper domain, a DNA-binding domain, and a nuclear localization signal. Once translocated to the nucleus, ATF6f activates the expression of UPR-related genes by binding to specific DNA elements, such as the cyclic AMP response element (CRE) and the ER stress response element (ERSE)^31^,^32^.

The human genome encodes two ATF6 paralogs—ATF6α and ATF6β^32^. Despite their high sequence similarity, ATF6β exhibits markedly reduced capacity to induce UPR-related gene expression due to a deficiency in its transcriptional activation domain. Studies indicate that ATF6β can form heterodimers with ATF6α, thereby exerting a transcriptional repressive effect^33^. In addition to modulating core UPR genes, ATF6 interacts with other transcription factors—such as cAMP response element-binding protein (CREB) and sterol regulatory element-binding proteins (SREBPs)—to synergistically amplify the UPR signaling cascade^34^,^35^. Through its cooperative actions with the IRE1 and PERK pathways, ATF6 enhances ER protein-folding capacity by upregulating the expression of molecular chaperones (e.g., GRP78) and folding enzymes (e.g., protein disulfide isomerase, PDI), thereby effectively alleviating ER stress^36^,^37^. However, under prolonged ER stress, ATF6 can also cooperate in the activation of pro-apoptotic genes such as CHOP, ultimately triggering programmed cell death^38^,^39^.

## 3. Physiological ER stress

The ER serves as a central hub for protein folding, lipid synthesis, and calcium homeostasis. Under physiological conditions, transient ER stress activates the UPR, a conserved adaptive mechanism that coordinates cellular growth, differentiation, metabolism, and stress resilience^40^-^42^. This section explores the dual role of physiological ER stress in maintaining cellular homeostasis and driving critical biological processes. The UPR, mediated by sensors PERK, IRE1, and ATF6, dynamically regulates cell fate by balancing protein synthesis, redox equilibrium, metabolic adaptation, autophagy, and mitochondrial function.

### 3.1. Cell growth and differentiation

Cellular differentiation and maturation in different cell types are often accompanied by an increase in protein synthesis, which can lead to the activation of ER stress^40^,^43^-^45^. The transient activation of the UPR can, in some cases, be crucial for achieving the morphological changes necessary for optimal cellular function, acting as a protective response. In mouse models, deletion of the PERK gene results in the loss of pancreatic β-cell structure without causing cell death, while promoting increased β-cell proliferation. This morphological alteration leads to a diabetic-like pathology, rather than the previously proposed mechanism of heightened cell death^46^.

Several hematopoietic lineages also rely on UPR activation to manage ER stress induced by immunoglobulin production and lysosomal compartment maturation, facilitating their proper differentiation^43^,^45^. The activation of UPR plays a pivotal role in the survival of these cells, with cell differentiation being a central aspect. For instance, the differentiation of B lymphocytes into plasma cells involves extensive ER expansion, and genetic modifications that drive immunoglobulin production underscore the importance of ER signaling in normal cellular processes^45^. Furthermore, studies have shown that UPR, specifically the PERK pathway, is crucial in maintaining the integrity of the hematopoietic stem cell pool under stress conditions, preventing functional deterioration^47^. Fibroblasts in the skin, which are responsible for collagen and matrix metalloproteinase production, exemplify how ER stress can influence cellular morphological changes. Their differentiation into myofibroblasts highlights how physiological ER stress contributes to such transformations^40^. The expression of old astrocyte specifically induced substance (OASIS) in osteoblasts is regulated by osteogenic factors such as bone morphogenetic protein 2 (BMP2), suggesting an upregulation of the PERK pathway during osteoblast differentiation. In this context, ATF4 compensates for defects in PERK-deficient osteoblasts and contributes to apoptosis regulation in bone remodeling^48^. This signaling network extends beyond bone physiology, influencing UPR signaling in astrocytes and regulating the differentiation of goblet cells in the colon across developmental stages^49^,^50^.

### 3.2. Oxidative stress

Oxygen-dependent cells are capable of producing reactive oxygen species (ROS). The cellular antioxidant defense mechanism substantially curtails ROS generation by directly interfering with free radical chain reactions and via detoxification enzymes such as superoxide dismutase and catalase, which facilitate the production of peroxidase^51^. The genesis of ROS is contingent upon various enzymes, including nicotinamide adenine dinucleotide phosphate-oxidase (NADPH, which conveys electrons to molecular oxygen), xanthine oxidoreductase, peroxidase, and the mitochondrial electron transport chain^51^. Oxidative stress arises when there is an imbalance between ROS production and the capacity of antioxidant defenses^52^. ROS act as a key link between ER stress and oxidative stress^42^. Both stressors are associated with cell death, which is triggered by changes in mitochondrial permeability, autophagy dysfunction, and inflammatory pathways^53^. ROS can directly engage the nuclear factor-kappa B (NF-kB) pathway, thereby augmenting the transcription of pro-inflammatory cytokines. The growth arrest and GADD34 is a direct downstream target of CHOP and can amplify ROS production within cells by enhancing protein synthesis^54^,^55^. The ER oxidoreductase 1 alpha (ERO-1α) is indispensable for the formation of disulfide bonds, assisting in protein folding and electron transfer to molecular oxygen, thus fostering the oxidation of ER-resident proteins. CHOP can augment ERO-1α expression, culminating in apoptosis^55^. Elevated ROS levels can increase intracellular Ca^2+^ concentrations, triggering apoptosis through the activation of the ER calcium channel inositol 1,4,5-trisphosphate receptor type 3 (ITPR3).

### 3.3. Metabolism

#### 3.3.1. Glucose metabolism

The UPR is pivotal in regulating glucose metabolism. Studies have indicated that the PERK-eIF2α pathway disrupts insulin signaling by modulating β-cell differentiation^46^. This pathway modulates both glucose and lipid metabolism via the transcription factors C/EBPα and C/EBPβ, which directly regulate glucose production and peroxisome proliferator-activated receptor gamma (PPARγ) activity^56^-^58^. Investigation has identified that the regulated IRE1-dependent RNA decay (RIDD) activity of IRE1 is responsible for decreasing the mRNA levels of proinsulin processing enzymes, such as insulin 1 (INS1), prohormone convertase 1 (PC1), and synaptophysin (SYP). These effects occur regardless of the presence of XBP1 or widespread UPR activation, underscoring the multifaceted role of IRE1 RNase activity^59^,^60^. Furthermore, IRE1α is integral to glucose sensing, with phosphorylation of IRE1α in response to physiological glucose levels enabling its regulation of insulin secretion. Glucose fluctuations trigger phosphorylation of IRE1α at Ser724, independent of the conventional activation pathway, and without triggering XBP1 mRNA splicing, JNK phosphorylation, or GPR78 dissociation from IRE1α^61^. On a molecular level, low glucose conditions promote binding of IRE1α to the adaptor protein receptor for activated C kinase 1 (RACK1), facilitating recruitment of the phosphatase protein phosphatase 2A (PP2A), thereby reducing IRE1α phosphorylation at Ser724^62^. In contrast, ER stress or acute glucose exposure disrupts the RACK1-PP2A complex, resulting in IRE1α activation through enhanced phosphorylation^62^. These findings highlight a dynamic regulatory system that modulates IRE1α phosphorylation in response to variations in glucose levels and ER stress.

#### 3.3.2. Lipid metabolism

The ER plays a critical role in maintaining lipid balance^63^. Hepatocytes, with their abundant smooth ER, are integral not only in protein synthesis but also in the production of bile acids, cholesterol, and phospholipids^64^. Liver-specific deletion of XBP1 in mice results in hypolipidemia, a phenomenon linked to the absence of XBP1-mediated feedback activation of IRE1. This activation of IRE1 triggers the degradation of mRNA encoding various genes involved in lipid metabolism via the RIDD pathway, underscoring the pivotal role of the IRE1-XBP1 signaling axis in lipid regulation^65^. Therefore, targeting XBP1 could present a promising therapeutic strategy for managing dyslipidemia. Furthermore, studies have highlighted XBP1's involvement in the synthesis and secretion of very low-density lipoproteins (VLDL) in IRE1 knockout mice with liver-specific alterations^66^. ATF6 has been associated with adipogenesis through the upregulation of genes involved in lipid synthesis and storage. The cleavage of ATF6 mirrors that of SREBPs, key regulators of lipid metabolism. In hepatocytes, the cleaved form of ATF6 interacts with SREBPs, recruiting histone deacetylase 1 (HDAC1) to inhibit the transcriptional activity of SREBPs^67^. Additionally, ATF6 plays a role in fatty acid oxidation through its interaction with PPARα^68^. XBP1s activates choline cytidylyltransferase, a rate-limiting enzyme in the cytidine diphosphate (CDP)-choline pathway, suggesting that IRE1/XBP1-driven lipid biosynthesis is crucial for lipid homeostasis^69^. Moreover, IRE1/XBP1 modulates both fatty acid synthesis and β-oxidation by indirectly activating PPARα^70^. In addition, fatty acid transport proteins (FATPs), which are substrates of RIDD, undergo mRNA degradation upon IRE1/RIDD pathway activation^71^. FATP contributes to lipid droplet formation, which, under physiological conditions, supports central neuronal homeostasis, yet may lead to neuronal damage in pathological states^72^. Hence, understanding the regulation of lipid droplet metabolism by FATP-mediated UPR signaling is vital.

#### 3.3.3. Amino acid metabolism

ER stress plays a significant role in amino acid metabolism, primarily through various branches of the UPR. ATF4, a key transcription factor in the UPR, mediates increased amino acid uptake under conditions of glutamine deficiency to maintain cellular amino acid homeostasis^73^. Additionally, a low-protein diet triggers an intracellular stress response, activating IRE1 and retinoic acid-inducible gene (RIG1), which subsequently upregulate cytokines that can induce anti-cancer immune responses in tumors^74^.

#### 3.3.4. ERAD

ERAD constitutes a crucial component of the ER-mediated protein quality control system, tasked with the correction of protein misfolding and the removal of aberrant proteins residing in the ER membrane or cytoplasm. The ERAD process can be categorized into four key stages: (1) recognition of misfolded proteins by molecular chaperones and lectins, (2) translocation across the ER membrane facilitated by valosin-containing protein (VCP), (3) polyubiquitination by E3 ubiquitin ligases, and (4) proteolysis by the 26S proteasome^75^. The terms ERAD-L, ERAD-M, and ERAD-C refer to substrates located in the ER lumen, membrane, and cytoplasm, respectively, which harbor folding defects or degradation signals^76^. ERAD is crucial for alleviating ER stress. Prolonged UPR activation, however, can impair protein synthesis, further aggravating ERAD dysfunction. Specifically, ER stress modulates eIF2α phosphorylation, which in turn regulates protein synthesis. This cascade can activate ATF4/CHOP, leading to increased protein synthesis and triggering apoptosis^55^. CHOP encodes a regulatory component of the eIF2α-directed phosphatase complex, facilitating the restoration of protein synthesis during cellular stress. Upon activation, the ATF6 pathway releases ATF6f, which directly regulates genes that encode proteins involved in the ERAD process, including Derlin-3^77^,^78^. The IRE1/XBP1 pathway ensures proper protein folding, maturation, and degradation within the ER, while also encoding chaperones like ER-localized DnaJ homolog 4 (ERdj4), 58 kDa inhibitor of protein kinase (p58IPK), ER degradation-enhancing alpha-mannosidase-like protein (EDEM), ribosome-associated membrane protein 4 (RAMP-4), PDI-P5, and Hsp40 ER-associated DnaJ homolog (HEDJ)^79^.

### 3.4. Autophagy

The ER stress modulates autophagy via signaling pathways, including AMP-activated protein kinase (AMPK), Protein Kinase B(AKT1)-mechanistic target of rapamycin(mTOR), and MAPK8^80^. Specifically, the ER and mitochondria play roles in ER stress, with the ER contributing to autophagosome formation through its expanded membrane. To counteract protein aggregation, ATF4 triggers reticulophagy by enhancing the interaction of ER surface proteins like cell-cycle progression gene 1 (CCPG1) and ATF8^81^,^82^. The DDRGK domain containing 1 (DDRGK)-dependent UFMylation of these proteins is suppressed by preceding ER stress during reticulophagy^83^. In age-associated disorders, the timely elimination of damaged mitochondria via mitophagy is vital for cellular survival. The PTEN-induced putative kinase 1 (PINK1)/Parkin pathway, which is central to mitophagy, can be downregulated by eIF2α/ATF4 gene knockout^84^. The eIF2α/ATF4 pathway is key to the transcription of autophagy-related genes such as p62, neighbor of BRCA1 gene 1 (NBR1), autophagy-related protein (ATG5), ATG7, ATG10, and gamma-aminobutyric acid receptor-associated protein (GABARAP) under ER stress^85^. In mammals, oligomeric IRE1 not only cleaves XBP1 mRNA but also activates stress-induced JNK by impeding autophagy, which in turn interacts with ASK1. Autophagy inhibition facilitates the binding of IRE1 to TRAF2, stabilizing its conformation and enabling interaction with ASK1. This indicates that the IRE1-ASK1-JNK axis is activated during pro-apoptotic signaling^86^,^87^. ER stress-induced autophagy may have detrimental effects. Prolonged ER stress activates all three branches of the UPR, leading to cell death via a complex involving pro-caspase-8, Fas-associated protein with death domain (FADD), and other components. This form of apoptosis is not dependent on mitochondria but requires ATG5, suggesting the involvement of autophagy^88^.

### 3.5. Mitochondrial dysfunction

Alterations in mitochondrial fusion, membrane permeability, transitions, pore formation, and dynamics can signify mitochondrial dysfunction, subsequently activating the NOD-like receptor protein 3 (NLRP3) inflammasome, intrinsic apoptosis, oxidative stress, and ER stress^89^. Research indicates a potential linkage between the outcomes of ER stress and mitochondrial fusion. The ER and mitochondria are in close association, working together to regulate lipid and calcium homeostasis. The region of contact between the ER and mitochondrial membranes is termed the mitochondria-associated ER membrane (MAM)^90^. Disruptions in either the ER or mitochondria can impact the other and elicit cellular responses. Upon ER stress, the activation of inositol trisphosphate receptor (IP3R) promotes the transfer of Ca2+ between the ER and mitochondria, which in turn triggers NLRP3 inflammasome activation^91^. Mitochondrial Ca2+ release can trigger ER stress. This implies that MAMs act as a conduit between NLRP3-induced inflammation and ER stress. Mitofusin 2 (MFN2) acts as an upstream regulator, inhibiting the activation of PERK and functioning as a critical link between mitochondrial metabolism and the UPR^92^. In melanoma, XBP1 enhances the ubiquitination and degradation of MFN2, promoting mitochondrial fission and mitophagy in response to ER stress^93^. Upon activation, CHOP significantly reduces Bcl-2 expression, while BH4-Tat mitigates mitochondrial membrane potential loss under ER stress, increases pro-apoptotic Bim levels, and activates caspases (including caspase-9, -2, and -3)^94^. This process leads to mitochondrial outer membrane permeabilization and the release of cytochrome c^88^. Furthermore, Bcl-2 influences the expression of Bcl-2 Homology 3 (BH3) domain-only proteins such as Bcl-2-associated X protein (BAX) and Bcl-2 homologous killer (BAK), which interact with mitochondria to promote membrane permeabilization^88^,^94^. As a result, CHOP orchestrates mitochondrial dysfunction and mitochondria-dependent intrinsic apoptosis through the Bcl-2, Bim, and caspase signaling pathways.

## 4. The stressors of ER stress in TME

**Figure 2:**
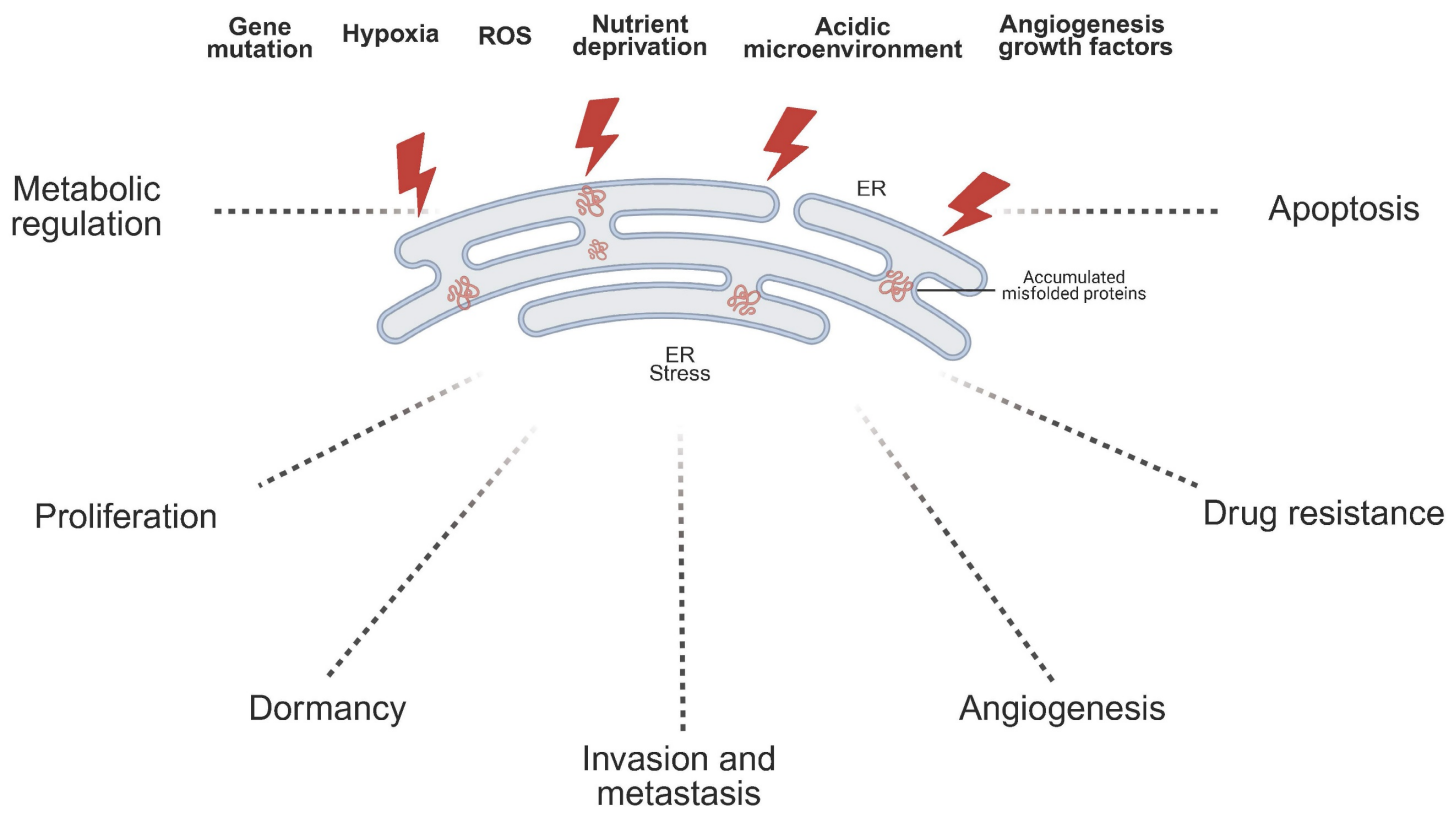
** The role of ER stress in cancer progression.** ER stress, induced by factors such as gene mutations, hypoxia, reactive oxygen species (ROS), nutrient deprivation, acidic microenvironment, and angiogenesis growth factors, leads to the accumulation of misfolded proteins in the ER. This stress can trigger various cellular responses, including apoptosis, metabolic regulation, drug resistance, proliferation, dormancy, invasion, metastasis, and angiogenesis. The adaptation to ER stress plays a significant role in tumor development and therapeutic resistance.

In the TME, the activation of ER stress is modulated by a variety of factors. These factors, commonly known as ER stressors, can trigger the UPR within the ER, thereby influencing tumor cell survival, proliferation, and invasiveness^95^ (Fig.2). ER stressors in the TME include genetic mutations, hypoxia, ROS, nutrient deprivation, and an acidic microenvironment. By disrupting ER homeostasis through distinct mechanisms, these stressors lead to the accumulation of unfolded proteins and the subsequent activation of UPR signaling pathways^95^,^96^.

### 4.1. Intracellular Stressors

Malignant tumor initiation and progression arise from either the inactivation of tumor suppressor genes or the acquisition of oncogenic mutations, a process that frees cell proliferation from the constraints of growth factor-dependent regulation. This transformation is frequently accompanied by an upregulation in protein synthesis that may exceed the folding capacity of the ER, thereby inducing ER stress^96^. Highly secretory tumors, such as multiple myeloma, are especially prone to sustained ER stress due to their extensive protein production^97^. Moreover, biological processes like epithelial-mesenchymal transition (EMT) further exacerbate ER stress by modulating the rate of protein synthesis^98^.

Missense mutations, by disrupting protein folding stability, can directly trigger ER stress when the capacity of the ER chaperone system is overwhelmed. In tumors with a high mutational burden, such as melanoma and lung cancer, these genetic alterations promote the activation of the UPR^99^. Typical oncogenic mutations—such as those in the Harvey rat sarcoma virus gene (HRAS, G12E) and the B-Raf proto-oncogene (BRAF, V600E)^10^,^11^—as well as deletions of tumor suppressor genes like p53 and Phosphatase and tensin homolog (PTEN)^100^,^101^, can significantly enhance protein synthesis, thereby intensifying ER stress responses. Notably, reducing the rate of protein translation can effectively alleviate ER stress and tumorigenesis^102^.

However, it is important to note that oncogene expression does not necessarily lead to ER stress. For example, in B-cell lymphomas, high c-MYC expression can protect tumor cells from ER stress-induced damage under conditions of proline deprivation^103^. This suggests that cells harboring oncogenic mutations may adapt to stress by modulating their ER protein folding capacity^104^.

### 4.2. Microenvironmental stressors

#### 4.2.1. Hypoxia

During tumor progression, microcirculatory dysfunction frequently leads to the establishment of hypoxic conditions within the TME. Hypoxia, as a critical stressor, can trigger ER stress by disrupting ER homeostasis^105^. The primary mechanism by which hypoxia induces ER stress involves the impairment of disulfide bond formation. Although disulfide bond formation is not strictly dependent on molecular oxygen, the precise post-translational folding and isomerization of proteins require an oxygen-rich environment. Under hypoxic conditions, the oxygen-dependent activity of ER Oxidoreductin 1 alpha (ERO1α), which catalyzes disulfide bond formation, is compromised, leading to protein misfolding and subsequent activation of the UPR^106^. Furthermore, hypoxia exacerbates ER stress by inhibiting lipid desaturation, thereby restricting the expansion of the ER membrane^107^.

The UPR activated via pathways such as PERK and IRE1 under hypoxic conditions is crucial for maintaining tumor cell survival. Once activated, PERK phosphorylates eIF2α, suppressing overall protein synthesis while upregulating ATF4 to enhance cellular tolerance to hypoxia^108^,^109^. Moreover, the IRE1-XBP1 branch of the UPR plays a pivotal role in hypoxia-mediated tumor growth. Studies have demonstrated that tumors deficient in XBP1 exhibit significantly reduced survival under hypoxic conditions and markedly diminished tumorigenic potential in vivo. Conversely, exogenous expression of spliced XBP1 can restore the tumor growth phenotype, underscoring the central regulatory role of this pathway in tumor progression^110^.

#### 4.2.2. ROS

The protein folding process within the ER is regulated by its redox state, which is intimately linked to the dynamic balance of ROS. Both extracellular stimuli and intracellular signaling events that lead to ROS accumulation can significantly disrupt ER protein homeostasis^111^. The underlying mechanism primarily involves the inhibition of glutathione biosynthesis—a critical antioxidant in the ER—thereby exacerbating oxidative stress and disturbing the redox equilibrium within the ER lumen^112^. Notably, mitochondrial metabolism also produces ROS as byproducts. These ROS further aggravate protein misfolding in the ER by modulating the function of ER-associated calcium channels and inducing lipid peroxidation reactions^113^, in addition to forming stable complexes with molecular chaperones^114^. Accumulated ROS in the ER can impair ER calcium homeostasis; increased cytosolic calcium levels not only impose additional functional burdens on the ER but also promote further ROS generation via mitochondrial pathways^115^. In this context, ROS signaling and the UPR establish a synergistic regulatory network that orchestrates the cellular stress response. Among these pathways, ER stress exerts a critical influence by activating signaling branches such as PERK. The PERK-mediated cascade, through the transcription factor nuclear factor erythroid 2-related factor 2 (NRF2), alleviates oxidative DNA damage and promotes cell proliferation—mechanisms that are closely associated with tumorigenesis and cancer progression^116^.

#### 4.2.3. Nutrient deprivation

Within the TME, nutrient deficiency serves as a critical trigger for ER stress. Specifically, the lack of glucose or glutamine inhibits the hexosamine biosynthetic pathway, leading to reduced production of uridine diphosphate-N-acetylglucosamine (UDP-GlcNAc), which is essential for proper protein folding in the ER^117^,^118^. Additionally, glucose deprivation suppresses the activity of the sarco/ER calcium ATPase (SERCA), resulting in disrupted ER calcium homeostasis and further contributing to ER stress^119^. It is noteworthy that the UPR key regulator, XBP1, is involved in the cellular adaptation to glucose deficiency. For instance, glucose restriction has been shown to induce XBP1 splicing and activation in primary breast cancer models, highlighting its central role in metabolic stress adaptation^120^. Furthermore, amino acid depletion activates general control nonderepressible 2 (GCN2), which phosphorylates eIF2α and initiates the integrated stress response (ISR). This ISR signaling pathway is not only a vital adaptive mechanism but also plays a significant role in the metabolic reprogramming of tumor cells^121^.

#### 4.2.4. Acidic microenvironment

Tumor cells predominantly utilize aerobic glycolysis for energy metabolism, a phenomenon termed the Warburg effect^122^. This metabolic reprogramming enables tumor cells to generate lactate, thereby lowering the pH of the surrounding TME. The acidic microenvironment, a hallmark of tumors, plays a critical role in promoting tumor survival and progression. Low pH conditions can elicit a variety of cellular responses through proton-sensing receptors, including the activation of ER stress via mechanisms such as disruption of intracellular calcium homeostasis and/or excessive production of ROS, which facilitates cellular adaptation to metabolic stress^123^,^124^. Within the acidic microenvironment, ER stress not only contributes to cellular adaptive regulation but also influences cell fate decisions by modulating key signaling pathways. Research has shown that low pH conditions can significantly alter the expression profile of Bcl-2 family proteins and activate the pro-apoptotic protein CHOP^125^. These regulatory mechanisms enable tumor cells to sustain survival by upregulating pro-survival signals under moderate stress, while triggering apoptosis under extreme stress conditions.

#### 4.2.5. Angiogenesis growth factors

The growth of solid tumors is constrained by an inadequate supply of oxygen, glucose, and other essential nutrients, particularly in the central regions where tumor cells exhibit rapid proliferation. To mitigate this nutrient and oxygen deprivation, tumor cells activate multiple pro-angiogenic pathways, including the secretion of vascular endothelial growth factor (VEGF) and fibroblast growth factor 2 (FGF2), which facilitate the formation of new blood vessels and support sustained tumor expansion. These stress conditions also trigger ER stress^126^. Existing research has demonstrated that various growth factors can induce ER stress in non-tumor models. For instance, members of the platelet-derived growth factor (PDGF) family, such as PDGF-A and PDGF-B, have been shown to elicit ER stress in experimental models of vascular injury, renal fibrosis, and lens development^127^. Consequently, the contribution of growth factors to tumor progression may be partially attributed to their capacity to activate the UPR.

Similarly, FGFs play a role in modulating ER stress and UPR pathways. For example, FGF-2 confers protection to cancer cells against ER stress-induced apoptosis through a mechanism involving Nck1, a protein associated with Src homology 2/3 domain signaling^128^. VEGF also contributes to the activation of the UPR. Specifically, VEGF signaling engages pathways involving phospholipase Cγ (PLCγ) and mTOR complex 1 (mTORC1), underscoring the critical roles of these components within the VEGF signaling cascade^129^. Notably, the UPR can further regulate VEGF signaling by preventing VEGF degradation via pathways mediated by IRE1α and ATF6^130^. Additionally, the UPR functions as a precursor regulator of VEGF transcription, exerting a direct influence on angiogenesis. Thus, the activation of the UPR serves not only as a cellular response to stress but also as a key regulator of growth factor signaling and angiogenesis—processes fundamental to tumor progression^126^. This highlights the intricate interplay among metabolic stress, UPR activation, and angiogenesis within the TME.

## 5. The multifaceted roles of ER stress in dictating tumor cell fate and behavior

The response to ER stress, known as the UPR, acts as a "double-edged sword" in cancer biology. The outcome of UPR activation is highly context-dependent, varying with the duration and amplitude of the stress, as well as the specific cellular environment. On one hand, severe or prolonged ER stress can trigger apoptosis, functioning as a crucial tumor-suppressive mechanism. On the other hand, adaptive UPR signaling is often exploited by cancer cells to promote their survival and progression. In this tumor-promoting role, the UPR influences a range of biological processes, including metabolic reprogramming, proliferation, dormancy, invasion, metastasis, and resistance to therapeutic agents^95^,^96^,^131^. These adaptive processes not only enhance the viability of tumor cells within challenging microenvironments but also exert significant effects on overall tumor progression and therapeutic outcomes.

### 5.1. Metabolic regulation

**Figure 3:**
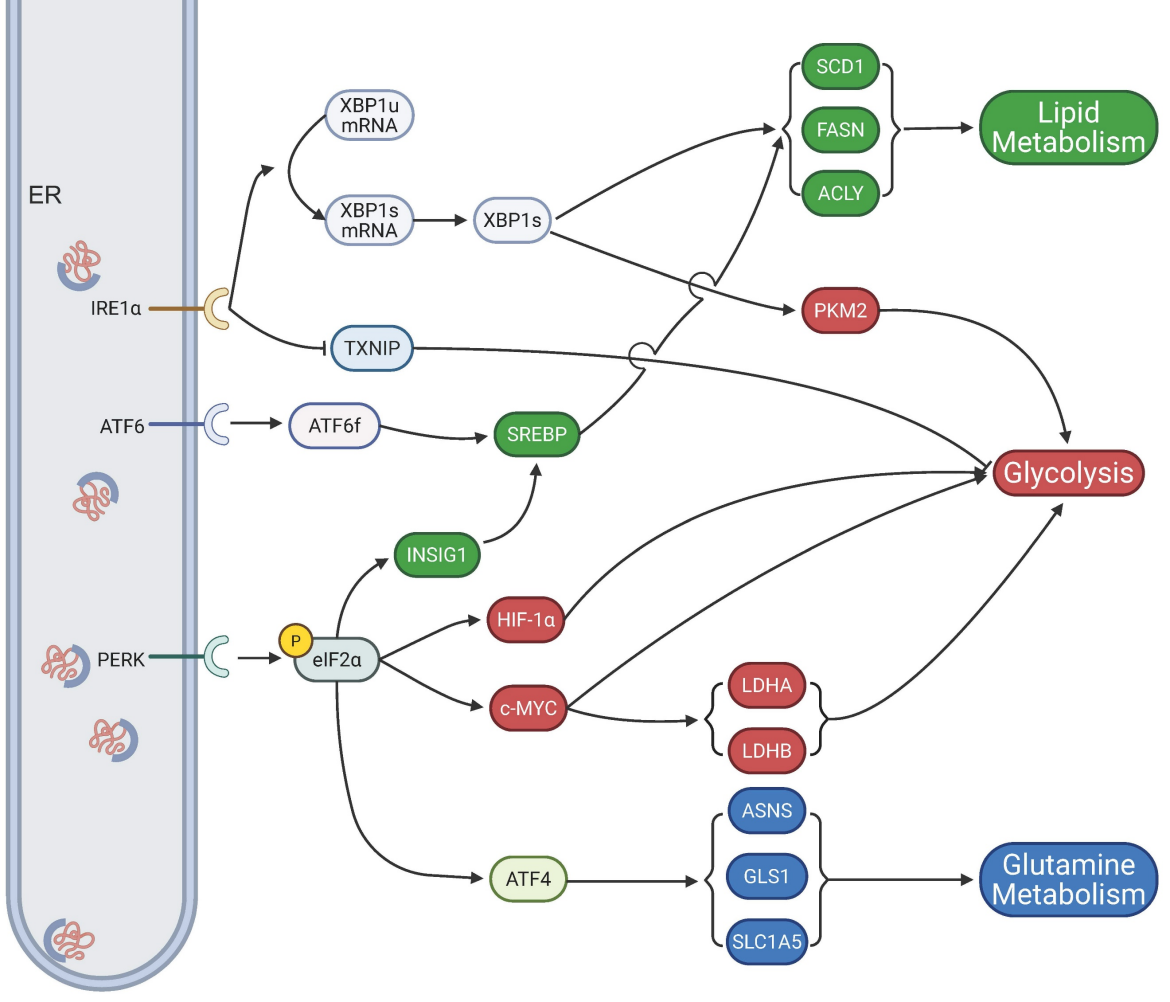
** ER stress-mediated regulation of tumor cell metabolism.** ER stress reprograms cellular metabolism to support tumor growth through the three canonical pathways of the UPR, initiated by the sensors PERK, IRE1α, and ATF6. Upon activation, the PERK pathway phosphorylates eIF2α, which enhances the translation of HIF-1α and c-MYC to drive glycolysis, and activates transcription factor ATF4 to promote glutamine metabolism by upregulating genes such as SLC1A5, ASNS, and GLS1. Concurrently, the IRE1α pathway, through the splicing of XBP1 mRNA to its active form XBP1s, upregulates genes involved in both lipid metabolism (SCD1, FASN, ACLY) and glycolysis (PKM2), while also suppressing the glycolysis inhibitor TXNIP. The ATF6 pathway contributes by activating the transcription factor SREBP to further promote lipid synthesis. Collectively, these ER stress signaling networks converge to regulate glycolysis, lipid metabolism, and glutamine metabolism, highlighting the critical role of the UPR in sustaining the metabolic demands of cancer cells.

Normal cellular metabolism serves as a cornerstone for maintaining physiological homeostasis. In malignant tumors, the rapid proliferation and invasive properties of cancer cells lead to pronounced abnormalities in their metabolic processes. Research has established that ER stress significantly influences tumor growth by regulating the glycolytic pathway (Fig.3). In pancreatic ductal adenocarcinoma (PDAC), overexpression of the basic leucine zipper and W2 domains 1 (BZW1) protein functions as an adaptor for PERK, promoting the phosphorylation of eIF2α. This activation enhances the translation efficiency of hypoxia-inducible factor-1α (HIF-1α) and c-MYC proteins via an internal ribosome entry site (IRES)-dependent mechanism, thereby inducing the Warburg effect and accelerating PDAC cell proliferation^132^. Notably, ERO1L, through ER stress-dependent upregulation, also contributes to the Warburg effect, driving PDAC progression^133^. In HeLa cells, treatment with 2-deoxyglucose or thapsigargin triggers ER stress, which upregulates lactate dehydrogenase A (LDHA) and LDHB subunits, facilitating cellular adaptation to aerobic glycolysis and enhancing tumor proliferation^134^. Additionally, estrogen activates the IRE1 pathway of the UPR, suppressing thioredoxin interacting protein (TXNIP) expression, which amplifies the Warburg effect and promotes breast cancer cell proliferation^135^. Furthermore, low XBP1 expression inhibits aerobic glycolysis in prolactinomas by downregulating pyruvate kinase M2 (PKM2)^136^.

In addition to glycolysis, reprogramming of lipid metabolism constitutes another metabolic hallmark of cancer^137^. Different ER stress pathways distinctly influence lipid synthesis. PERK deficiency markedly reduces the expression of lipid biosynthetic enzymes, including fatty acid synthase (FASN), ATP citrate lyase (ACLY), and stearoyl-CoA desaturase 1 (SCD1), underscoring the importance of PERK signaling in maintaining lipogenic programs^138^. Similarly, activation of the IRE1α/XBP1s signaling axis promotes tumor growth and invasion by inducing SCD1 expression, thereby supporting fatty acid desaturation and membrane biosynthesis^139^,^140^. Additionally, crosstalk between ER stress and SREBPs has been reported: ATF6 and PERK signaling can modulate SREBP activation, thereby altering cholesterol and fatty acid synthesis. Importantly, when ER stress is severe and sustained, such as during apigenin exposure in HepG2 hepatocellular carcinoma cells, lipid metabolism-related enzymes are initially upregulated but progressively downregulated with prolonged stress, indicating that excessive ER stress disrupts lipid homeostasis through ER damage^141^.

Furthermore, amino acid metabolism—particularly glutamine utilization—is tightly coupled to ER stress. The PERK-eIF2α-ATF4 axis functions as a central mediator of the amino acid response during ER stress. ATF4 enhances the transcription of amino acid transporters and metabolic enzymes, including solute carrier family 1 member 5 (SLC1A5), glutaminase 1 (GLS1), and asparagine synthetase (ASNS), thereby increasing glutamine uptake and driving glutaminolysis^142^. This process replenishes tricarboxylic acid (TCA) cycle intermediates, supports nucleotide biosynthesis, and fuels glutathione (GSH) synthesis to maintain redox balance. Several studies have further shown that ATF4 activation under nutrient or ER stress conditions creates a dependency on glutamine for tumor cell survival and proliferation^143^,^144^. Moreover, ER stress-associated transcriptional regulators, such as high mobility group AT-hook 2 (HMGA2), can augment the expression of glutamine transporters and metabolic enzymes, thereby reinforcing glutamine addiction in cancer cells^145^,^146^. Collectively, these findings underscore the pivotal role of the UPR in coordinating glucose, lipid, and glutamine metabolism to sustain malignant growth.

### 5.2. Proliferation

ER stress plays a pivotal role in regulating tumor cell proliferation^147^. In hepatocellular carcinoma (HCC), tripartite motif-containing 25 (TRIM25) serves as a downstream effector of the ER stress response, specifically activating the IRE1-JNK signaling branch within the UPR signaling network. Furthermore, it promotes HCC cell proliferation by activating the ERAD pathway via the Kelch-like ECH-associated protein 1 (KEAP1)-NRF2 axis^148^. In melanoma, UPR activation drives tumor cell proliferation through the interleukin-6 (IL-6)/signal transducer and activator of transcription 3 (STAT3) signaling axis^149^. In non-small cell lung cancer (NSCLC), the upregulation of prolactin receptor-like protein 11 (PRL11) enhances tumor proliferation by modulating both the UPR and autophagy pathways^150^. Additionally, the IRE1-XBP1 signaling pathway has been implicated in MYC-driven tumor progression in breast cancer and urothelial carcinoma^151^. Studies further demonstrate that during ER stress, IRE1α recruits TRAF2 to activate the JNK and NF-κB signaling cascades, thereby promoting tumorigenesis^152^,^153^. Similarly, ATF6 contributes to malignant tumor proliferation. In colorectal cancer, phosphorylated ATF6 induces gut microbiota dysbiosis and activates the TRIF/STAT3 signaling pathway, accelerating tumor progression^154^. Moreover, ATF6 promotes cervical cancer cell proliferation via the MAPK pathway^155^.

### 5.3. Dormancy

Studies have demonstrated that the UPR plays a critical role in regulating dormancy in malignant tumor cells. By enabling tumor cells to adapt to the harsh microenvironments of distant organs, the UPR promotes the survival of disseminated tumor cells (DTCs) in a dormant state^156^-^158^. Notably, in breast cancer patients, DTCs within the bone marrow exhibit elevated expression of the UPR-associated proteins BiP and GRP94, suggesting their potential involvement in maintaining tumor cell dormancy^158^. Further investigations have revealed that the activation of IRE1α is essential for inducing the dormant phenotype in tumor cells, a process mediated by the activation of p38, a key regulator of dormancy in human malignant tumors^159^. Additionally, the PERK pathway contributes to dormancy regulation by downregulating the translation of critical cell cycle regulators, thereby inducing G0-G1 phase arrest^157^,^160^. In PDAC, the PERK pathway is significantly overactivated in DTCs located in the liver^161^. In dormant squamous cell carcinoma, the nuclear translocation of ATF6 enhances the ability of tumor cells to withstand stresses such as chemotherapy and nutrient deprivation. This is achieved through the activation of the AKT-independent mTOR signaling pathway, which supports the survival of quiescent cells in adverse microenvironments^156^,^159^,^162^. Collectively, these findings highlight the pivotal role of sustained ER stress in determining the fate of dormant tumor cells.

### 5.4. Invasion and metastasis

ER stress plays a central role in driving tumor invasion and metastasis through its regulation of EMT. EMT is a critical biological process whereby epithelial cells undergo phenotypic transformation to acquire mesenchymal characteristics, thereby enhancing their migratory and invasive capabilities^163^. This transformation is facilitated by the UPR, which acts in concert with extracellular signaling pathways such as ERK/MAPK and PI3K/AKT to upregulate key EMT transcription factors^164^. In cervical cancer models, ATF6 has been shown to induce EMT by suppressing E-cadherin expression while increasing the levels of mesenchymal markers such as Snail^155^. Similarly, in pancreatic cancer cells, calreticulin enhances ER stress-induced EMT by upregulating the transcription factor Snail2 and activating the ERK pathway^165^. Notably, the UPR also contributes to the formation of the pre-metastatic niche, a process that involves remodeling the microenvironments of distant organs to support tumor colonization^166^,^167^. For instance, in salivary adenoid cystic carcinoma (SACC), extracellular vesicles derived from highly metastatic SACC cells, modified by α2,6-sialylation, activate the PERK-eIF2α pathway. This activation disrupts vascular endothelial cadherin expression, increases vascular permeability, and promotes lung metastasis, a process closely linked to the formation of polymorphonuclear neutrophils (PMN)^166^,^167^. Additionally, during the metastatic cascade, tumor cells must evade anoikis to survive in the circulation and establish secondary tumors. The PERK signaling axis confers a survival advantage to metastatic cells by inhibiting anoikis^168^,^169^. In cells undergoing EMT, PERK activation not only boosts their secretory functions but also helps maintain their invasive phenotype^98^. Importantly, metastatic cells often experience heightened oxidative stress compared to primary tumor cells, necessitating metabolic reprogramming, including the synthesis of antioxidants, for survival^170^. The PERK branch of the UPR mitigates oxidative stress and supports metastatic growth by modulating ATF4 and NRF2-mediated antioxidant responses^171^.

### 5.5. Angiogenesis

ER stress plays a pivotal role in modulating the transcriptional and post-translational expression of various angiogenesis-related factors^126^. Specifically, the IRE1 pathway of the UPR has emerged as a critical regulator of tumor angiogenesis. In malignant gliomas, IRE1 governs the expression of pro-angiogenic factors, including VEGF-A, IL-6, and IL-8^172^. Suppression of IRE1α in these tumors results in the downregulation of these pro-angiogenic cytokines, consequently impairing tumor angiogenesis and metastasis. Additionally, the IRE1 pathway contributes to the stabilization of HIF-1α, a central regulator of angiogenesis under hypoxic conditions, further emphasizing its significance in promoting vascularization within solid tumors^172^. The intricate interplay between IRE1 and other molecular pathways, such as HIF-1, underscores the complexity of tumor angiogenesis. HIF-1α is widely recognized for its role in enhancing the expression of VEGF and other angiogenic factors during hypoxia^173^. Research has also demonstrated that XBP1, a downstream effector of IRE1, can facilitate angiogenesis independently of VEGF, indicating the presence of alternative pathways through which IRE1 regulates tumor vascularization^174^. In triple-negative breast cancer (TNBC), inhibition of IRE1α has been shown to disrupt cellular adaptation to ER stress, thereby augmenting the efficacy of anti-angiogenic therapies^175^.

Another essential arm of the UPR, the PERK pathway, similarly promotes tumor angiogenesis through downstream effectors such as ATF4, which enhances the production of angiogenic factors^126^,^176^. In glioma cells, PERK activation is associated with the upregulation of peptidylglycine α-amidating monooxygenase, a protein that supports angiogenesis and accelerates tumor growth^177^. Likewise, in PDAC, the PERK signaling cascade within cancer-associated fibroblasts (CAFs) drives endothelial-like transformation of CAFs and stimulates vessel formation, thereby fostering the development of tumor-associated vasculature^177^.

While ER stress and the UPR predominantly mediate pro-angiogenic effects, there are instances where ER stress exerts an inhibitory influence on angiogenesis. For example, tumor-secreted exosomes induced by ER stress can suppress angiogenesis, highlighting the nuanced role of ER stress in tumor vascularization^178^. Furthermore, low-intensity pulsed ultrasound has been shown to activate p38-mediated ER stress, triggering endothelial cell apoptosis and inhibiting angiogenesis, which suggests potential therapeutic applications of ER stress modulation in cancer treatment^179^.

### 5.6. Drug resistance

Chemotherapy resistance is a multifactorial phenomenon, typically resulting from the interplay of intrinsic and acquired mechanisms within tumor cells^180^. Recent research has established a strong correlation between the ER stress-activated UPR and resistance to chemotherapy, targeted therapy, and immunotherapy. ER stress has been shown to promote resistance, particularly in aggressive cancer types. The UPR influences tumor cell survival and chemosensitivity by triggering various downstream signaling cascades. For instance, ER stress activates the PERK and NRF2 pathways, leading to the upregulation of multidrug resistance-related proteins, such as multidrug resistance-associated protein 1, thereby fostering a chemotherapy-resistant phenotype in tumor cells^181^. Furthermore, ER stress enhances DNA repair mechanisms in tumor cells, increasing their capacity to withstand chemotherapy-induced damage^182^. In colon cancer, for example, ER stress upregulates the zinc finger protein 263-guanine nucleotide exchange factor 2 pathway, contributing to treatment resistance^183^. Another study found that in nasopharyngeal carcinoma, ER stress induces the secretion of exosomes containing ER protein 44 (ERP44), which transmit chemotherapy resistance to neighboring cells^184^. In pancreatic cancer, the UPR sensor GRP78 interacts with the extracellular domain of calcium phosphate binding protein 1-like (CLPTM1L)/cisplatin resistance-related protein 9 (CRR9), facilitating the development of chemoresistance^185^. The role of ER stress in drug resistance is further mediated by the activation of IRE1 and ATF6 signaling pathways. The IRE1 pathway modulates the expression of ABC transporters, altering drug efflux mechanisms and promoting resistance to agents such as 5-fluorouracil^186^. Similarly, ATF6 activation upregulates BRCA1, enhancing DNA repair capacity and leading to resistance against doxorubicin^187^.

Conversely, ER stress can also function as a double-edged sword by exerting pro-apoptotic effects to counteract chemotherapy resistance. Certain studies suggest that inducing ER stress can sensitize cancer cells to chemotherapy by activating cell death pathways. For example, the ER stress inducer tunicamycin has been demonstrated to enhance chemotherapy-induced apoptosis in multidrug-resistant gastric cancer cells^188^. Likewise, in breast cancer cells, betulinic acid activates ER stress, restoring sensitivity to chemotherapy^189^. In colorectal cancer, activation of PERK has been reported to increase sensitivity to paclitaxel, while the combination of 5-fluorouracil and withaferin A promotes apoptosis via the PERK axis, overcoming chemoresistance^190^. These findings highlight the potential of targeting ER stress pathways to resensitize cancer cells to chemotherapy, offering valuable insights for novel therapeutic approaches. In pancreatic cancer, downregulation of phosphoglucomutase 3 has been shown to activate the UPR, thereby enhancing sensitivity to gemcitabine^191^. This further underscores the possibility of modulating the UPR to reverse cancer drug resistance. Additionally, research has demonstrated that activation of the IRE1-XBP1 signaling pathway can overcome resistance to ibrutinib in diffuse large B-cell lymphoma^192^. These discoveries suggest that targeting ER stress may represent a promising therapeutic strategy for specific malignancies.

### 5.7. Apoptosis

Under conditions of prolonged or severe ER stress that surpass cellular capacity for restoring ER homeostasis, the resultant proteostatic failure triggers apoptotic pathways through multiple signaling cascades, particularly the IRE1/TRAF2/ASK1/JNK axis and CHOP pathway^4^,^193^-^197^. The IRE1/TRAF2/ASK1/JNK signaling cascade represents a principal apoptotic pathway during ERS. During ER stress, the transmembrane kinase IRE1 forms a complex with TRAF2, facilitating activation of ASK1. This activation initiates downstream JNK signaling, ultimately culminating in apoptotic execution^193^,^194^. Concurrently, the CHOP pathway becomes activated through nuclear translocation of transcriptional regulators including ATF4, ATF6, and XBP1. These factors enhance CHOP transcriptional activity in the nucleus, promoting upregulation of apoptotic effectors such as caspase-3 and establishing a pro-apoptotic signaling cascade^198^. The tumor-selective activation of this pathway has been documented across various malignancies including gastric and cervical carcinomas. Notably, phytochemicals like quercetin and shikonin demonstrate potent pro-apoptotic effects in cancer cells through activation of the IRE1-JNK-CHOP axis, highlighting therapeutic potential in oncological contexts^199^.

Emerging evidence implicates misfolded proteins as endogenous ligands for death receptors during ERS. Specifically, intracellular activation of DR5 by misfolded proteins occurs independently of its canonical extracellular ligand TNF-related apoptosis-inducing ligand (TRAIL)^200^. Counterregulatory mechanisms exist through the RIDD activity of IRE1α, which degrades DR5 mRNA to mitigate apoptosis^201^. Current models propose that cellular fate determination depends on the dynamic balance between PERK-mediated signaling and IRE1α-driven RIDD activity. However, the precise interplay of these pathways in malignant cells remains incompletely characterized and warrants further investigation.

## 6. ER stress in TME remodeling

**Figure 4:**
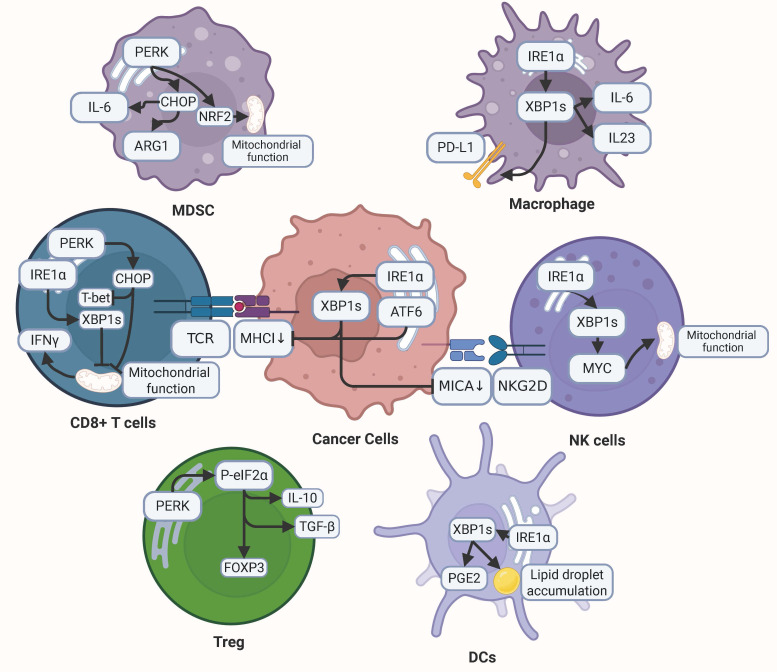
** ER stress in TME remodeling.** ER stress plays a pivotal role in shaping the TME by modulating the behavior of both cancer cells and immune cells, ultimately promoting immune evasion and tumor progression. In cancer cells, ER stress activates specific signaling pathways, such as IRE1α-XBP1s and ATF6, which downregulate MHC-I and MICA. This downregulation hinders recognition by CD8⁺ T cells and natural killer (NK) cells, respectively, enabling cancer cells to escape immune surveillance. Within the TME, ER stress impacts CD8⁺ T cells through the PERK and IRE1α-XBP1s pathways, suppressing the production of T-bet and IFNγ while impairing mitochondrial function, thus weakening their antitumor activity. Meanwhile, Tregs bolster their immunosuppressive effects via the PERK-eIF2α pathway, which upregulates Foxp3, IL-10, and TGF-β. Similarly, MDSCs enhance their immune-suppressive capacity by increasing IL-6 and ARG1 expression through the PERK-CHOP-NRF2 axis. In macrophages, activation of the IRE1α-XBP1s pathway drives the upregulation of PD-L1, IL-6, and IL-23, fostering a pro-tumorigenic milieu. DCs under ER stress, particularly via IRE1α-XBP1s signaling, accumulate lipid droplets and produce PGE2, compromising their antigen presentation capabilities. Additionally, NK cells rely on the IRE1α-XBP1s pathway to support MYC-mediated mitochondrial function, which is critical for their antitumor efficacy.

ER stress plays a central role within the TME, exerting dual regulatory influences on both malignant cells and their surrounding stromal and immune components (Fig.4). Accumulating evidence indicates that ER stress not only governs the intrinsic biological properties of tumor cells but also drives TME remodeling. Such remodeling, in turn, reshapes the immunological landscape, thereby dictating the responsiveness to immune checkpoint blockade and other immunotherapeutic strategies^202^,^203^. These processes operate through intricate mechanisms that ultimately modulate immune cell function and tumor progression^204^-^208^. A more comprehensive understanding of ER stress-mediated TME reprogramming is therefore of critical importance for unraveling the molecular complexity of tumor biology and for guiding the development of innovative therapeutic interventions targeting this highly dynamic cellular ecosystem.

### 6.1. ER stress in tumor cells

Emerging evidence suggests that ER stress in tumor cells may impair antigen presentation through destabilization of major histocompatibility complex class I (MHC-I) molecules via overexpression of XBP1s and ATF6^209^. Lymphoma studies demonstrate that under palmitate or glucose deprivation conditions, ER stress-mediated suppression of protein synthesis through eIF2α phosphorylation disrupts MHC-I-dependent antigen presentation^210^. Furthermore, ER stress has been shown to downregulate transporter associated with antigen processing 1 (TAP1), thereby impeding MHC-I peptide loading^211^. The activation of ER stress pathways significantly impacts T cell-mediated tumor surveillance. Pancreatic cancer models reveal that malignant cell-specific XBP1s overexpression promotes metastatic progression by attenuating T cell-mediated antitumor immunity^212^. In melanoma models, XBP1 inhibition potentiates immune checkpoint blockade efficacy, suggesting ER stress pathways may modulate therapeutic immune responses^213^.

Accumulating evidence indicates that tumor-associated ER stress influences natural killer (NK) cell surveillance mechanisms. The IRE1α-XBP1 signaling axis has been demonstrated to reduce expression of MHC class I polypeptide-related sequence A (MICA), a ligand for natural killer group 2D (NKG2D) receptors on NK cells, thereby compromising tumor recognition in melanoma^214^. ER stress also exerts profound effects on myeloid cell recruitment and function. ER-stressed cancer cells secrete factors capable of remote myeloid reprogramming. In chronic lymphocytic leukemia (CLL), malignant cells exploit the IRE1α-XBP1 pathway to overproduce secretory immunoglobulin M (sIgM), driving myeloid-derived suppressor cells (MDSCs) accumulation and enhancing their immunosuppressive activity^215^. Tumor-derived factors from ER-stressed prostate, lung, and melanoma cells induce pro-tumorigenic dendritic cell (DC) alterations, including upregulation of immunosuppressive arginase-1 and prostaglandin E2^216^. Notably, exposure to conditioned media from ER-stressed cancer cells confers DCs with immunosuppressive phenotypes that promote in vivo tumor growth^217^,^218^. Hepatocellular carcinoma models reveal that ER-stressed tumor cells release exosomal miR-23a-3p to enhance programmed death-ligand 1 (PD-L1) expression in macrophages, amplifying their CD8+ T cell suppressive capacity and correlating with poor patient prognosis^219^. Moreover, soluble factors secreted by ER-stressed tumor cells induce upregulation of proinflammatory cytokines that impair antigen-presenting cell functionality, creating an immunosuppressive TME^7^.

### 6.2. ER stress in immune cells

#### 6.2.1. T cells

ER stress induced by the TME plays a pivotal role in regulating T cell dysfunction. Studies demonstrate that malignant ascites from ovarian cancer patients suppresses glucose uptake and induces defective N-linked protein glycosylation in T cells, thereby activating the IRE1α/XBP1 signaling pathway which subsequently impairs mitochondrial activity and interferon-gamma (IFN-γ) production^220^. Notably, pharmacological blockade of the XBP1 pathway under nutrient-deprived conditions restores mitochondrial respiratory function in T cells both in vitro and in vivo, enhances IFN-γ secretion, and significantly potentiates antitumor immune responses^220^. Furthermore, the IRE1α-XBP1s axis-mediated immune checkpoint activation in CD8+ T cells promotes T cell exhaustion, whereas genetic ablation of XBP1 reverses this process, effectively restoring T cell functionality and prolonging survival^221^.

The PERK signaling pathway in tumor-infiltrating T cells contributes substantially to multiple mechanisms of tumor immune evasion. Elevated CHOP expression in CD8+ T cells within the TME correlates with poor clinical outcomes, primarily through CHOP-mediated downregulation of T-bet, a master transcription factor essential for antitumor immunity^222^. Additionally, CHOP activation compromises T cell metabolic fitness by simultaneously disrupting both glycolytic and mitochondrial metabolic pathways, thereby attenuating effector functions^222^. Intriguingly, while chronic activation of the PERK-CHOP axis induces metabolic dysregulation and immunosuppression, acute stress-induced PERK activation triggered by signals like carbon monoxide initiates mitophagy, restores mitochondrial integrity, and enhances antitumor responses in T cells^223^.

ER stress also critically regulates regulatory T cell (Treg) functionality. ER stress induction in Tregs significantly upregulates forkhead box P3 (Foxp3) expression while enhancing production of immunosuppressive cytokines IL-10 and transforming growth factor-beta (TGF-β). This immunoinhibitory effect is markedly attenuated upon pharmacological inhibition of the PERK-eIF2α signaling pathway^224^,^225^. These findings collectively suggest that ER stress-mediated enhancement of Treg immunosuppressive capacity within the TME may further compromise antitumor immune efficacy.

#### 6.2.2. Macrophages

As pivotal components of myeloid cell populations, macrophages exhibit functional regulation closely associated with the ER stress, with the IRE1α-XBP1 signaling axis demonstrating particular significance. This pathway not only governs macrophage polarization states but also modulates cytokine secretion profiles, particularly enhancing the release of pro-tumorigenic factors that facilitate cancer cell invasion^226^,^227^. Comparative studies reveal that mice with macrophage-specific IRE1α knockout show significantly prolonged tumor-bearing survival compared to wild-type controls, indicating the pathway's tumor-promoting role in cancer progression^226^. Mechanistically, lipid accumulation in macrophages activates ER stress pathways, subsequently triggering IRE1α-XBP1 signaling activation^228^. This dual process amplifies both the immunosuppressive functions of macrophages and their lipid storage capacity, collectively shaping a tumor-favorable TME.

Notably, TME-derived cytokines such as IL-4, IL-6, and IL-10 activate the IRE1α-XBP1 cascade through STAT3/STAT6 signaling pathways. This transcriptional reprogramming induces cathepsin secretion by macrophages, thereby potentiating their capacity to mediate tumor cell invasion^210^. The synergistic interaction between metabolic adaptation (lipid accumulation) and cytokine-mediated signaling establishes a self-reinforcing loop that sustains tumor-promoting macrophage activities within the TME.

#### 6.2.3. MDSCs

MDSCs, a heterogeneous population of immature myeloid cells with potent T-cell suppressive capacity, play a pivotal role in establishing the immunosuppressive TME^229^. The regulatory mechanisms governing MDSC functionality are centrally mediated by the ER stress, particularly through its PERK signaling axis. This pathway not only maintains mitochondrial DNA (mtDNA) integrity but also preserves the immunosuppressive properties of MDSCs across diverse tumor models. ROS and peroxynitrite prevalent in the TME activate the PERK pathway in MDSCs, thereby inducing elevated expression of IL-6 and arginase. This molecular cascade subsequently enhances MDSC accumulation and augments their T cell suppressive functions^230^.

PERK activation triggers phosphorylation and subsequent activation of NRF2, a master regulator of cellular redox homeostasis. This molecular event enables MDSCs to counteract oxidative stress, thereby sustaining their survival and immunosuppressive potency^171^. Pharmacological or genetic inhibition of PERK disrupts NRF2 signaling in MDSCs, leading to mitochondrial dysfunction and cytoplasmic release of mtDNA. These cellular alterations activate the stimulator of interferon genes (STING) pathway, inducing type I interferon responses that ultimately potentiate antitumor immunity. This mechanism significantly enhances the therapeutic efficacy of immune checkpoint blockade and adoptive T-cell therapy^171^.

Furthermore, genetic ablation of downstream PERK effectors (CHOP or ATF4) in tumor stroma substantially attenuates MDSC-mediated immunosuppression through downregulation of critical molecular mediators including C/EBPβ, phosphorylated STAT3, and IL-6^231^. Analogously, knockout of GCN2, a nutrient-sensing kinase responsible for eIF2α phosphorylation, suppresses ATF4 activation and consequently impairs the immunosuppressive functions of both MDSCs and tumor-associated macrophages (TAMs)^232^. Experimental evidence from CHOP knockout murine models and bone marrow transplantation studies with CHOP-deficient cells demonstrates significant retardation of tumor progression, confirming the central role of CHOP in MDSC-mediated immunosuppression.

Notably, activation of other UPR branches (IRE1α and ATF6) similarly enhances the immunosuppressive capacity of PMN-MDSCs^233^. Intriguingly, induction of ER stress in normal neutrophils can reprogram them into PMN-MDSCs with potent T-cell inhibitory properties. This phenotypic conversion is effectively blocked by inhibition of the RNase domain of IRE1α, suggesting that targeted modulation of ER stress pathways in MDSCs may offer novel therapeutic strategies for cancer immunotherapy^234^.

#### 6.2.4. Dendritic cells

The mechanism underlying ER stress-mediated DC dysfunction has emerged as a pivotal research focus in tumor immunology. Current investigations reveal that aberrant accumulation of ROS within TME-resident DCs triggers lipid peroxidation, subsequently activating the IRE1α-XBP1 signaling cascade. Sustained activation of this pathway initiates a pro-lipogenic transcriptional program, resulting in pathological cytoplasmic lipid droplet accumulation^114^,^235^. Experimental evidence from ovarian cancer murine models demonstrates that ROS accumulation in DCs facilitates generation of lipid peroxidation byproducts. These metabolites exacerbate ER stress through modulation of ER-resident chaperone activity, thereby perpetuating UPR-mediated IRE1α-XBP1 pathway activation and driving aberrant upregulation of triglyceride biosynthesis and lipid droplet formation genes^235^.

Notably, intracellular lipid accumulation serves as a hallmark of DC dysfunction, with resultant antigen presentation defects significantly impairing T cell activation within TME. Recent breakthroughs demonstrate that genetic ablation or pharmacological inhibition of IRE1α/XBP1 expression in DCs effectively reverses these pathological manifestations. Specifically, DC-specific IRE1α or XBP1 knockout models and RNA interference strategies markedly restore antigen-presenting capacity and enhance T cell-mediated antitumor immunity^235^. In preclinical ovarian cancer models, these interventions significantly delay tumor progression and improve survival outcomes, suggesting therapeutic targeting of the IRE1α-XBP1 axis in DCs could potentiate endogenous antitumor immunity and improve immunotherapy efficacy.

Furthermore, the IRE1α-XBP1 signaling axis exhibits dual regulatory functions beyond lipid metabolism, extending to immunomodulatory mediator production. Mechanistic studies confirm that pathway activation in DCs transcriptionally upregulates prostaglandin E2 (PGE2) biosynthetic enzymes. As a potent immunosuppressive lipid mediator, PGE2 exerts broad-spectrum inhibition of immune responses^236^. This finding elucidates the dual role of IRE1α-XBP1 signaling in coordinating both metabolic reprogramming and immunosuppressive microenvironment formation within TME, highlighting the multifaceted regulatory functions of DCs in tumor immunology.

#### 6.2.5. NK cells

The IRE1α-XBP1 signaling pathway plays an indispensable role in maintaining the proliferative capacity of NK cells^237^. Mechanistic investigations revealed that this pathway enhances mitochondrial respiratory capacity through MYC induction, a critical process for NK cell proliferation. In conditional knockout mouse models with NK cell-specific deletion of either IRE1α or XBP1, experimental groups demonstrated significantly impaired tumor immunosurveillance compared to wild-type controls following intravenous challenge with B16F10 melanoma cells. Specifically, these mice exhibited three hallmark pathological features: reduced NK cell infiltration into tumor sites, increased pulmonary metastatic nodules, and diminished host survival rates^237^. Furthermore, comprehensive flow cytometric analysis revealed impaired effector function in IRE1α-deficient NK cells, characterized by reduced granzyme B production and attenuated interferon-γ secretion. These findings collectively demonstrate that the IRE1α-XBP1 axis serves as a critical metabolic checkpoint governing NK cell homeostasis and anti-tumor competence.

## 7. Unconventional modulators of ER stress in cancer therapy

In addition to conventional therapeutic strategies, a significant body of research is focused on identifying unconventional modulators of ER stress for cancer therapy. These agents, derived from both natural products and targeted synthesis, offer novel mechanisms to induce tumor-selective cell death by targeting specific components of the UPR. This section delineates the therapeutic potential of two major classes of these modulators: organic compounds, including phytochemicals like berberine and flavonoids, and innovative metal-based coordination compounds. The discussion will highlight their unique modes of action and their promise in preclinical cancer models.

### 7.1. Organic compounds targeting ER stress

Berberine, an isoquinoline alkaloid isolated from plants such as Berberis vulgaris, exemplifies selective induction of ER stress^238^. It depletes ER Ca²⁺ stores, thereby activating the PERK/eIF2α/ATF4 and IRE1/XBP1 pathways, culminating in caspase-4-dependent apoptosis. In gastric and lung adenocarcinoma models, berberine demonstrates sub-micromolar IC₅₀ values, with toxicity attenuated by the PERK inhibitor salubrinal, confirming pathway specificity. Its notable features include cell line-agnostic activity and efficacy in 3D spheroid cultures, suggesting therapeutic potential against heterogeneous tumors. Moreover, berberine synergizes with cisplatin in NSCLC, augmenting ER stress-mediated radiosensitivity^239^.

Emodin, an anthraquinone derivative from rhubarb (Rheum palmatum)^240^, selectively perturbs cytosolic Ca²⁺ homeostasis in A549 lung cancer cells, leading to upregulation of UPR genes (ATF4, GRP78/BiP, CHOP) through PERK and IRE1 signaling. Unlike broad-spectrum inducers, emodin induces caspase-independent death in certain cell lines, implicating alternative modes such as paraptosis, while retaining potency in 3D tumor models. Its unconventional feature lies in lung-specific selectivity, sparing gastric epithelial cells and fibroblasts, thereby positioning it as a promising lead compound for pulmonary malignancies^239^.

Curcumin, a polyphenolic curcuminoid derived from turmeric (Curcuma longa), functions as an allosteric inhibitor of SERCA, promoting eIF2α phosphorylation and CHOP/DR5 upregulation to facilitate TRAIL-mediated apoptosis^241^. In ovarian cancer and cisplatin-resistant NSCLC, it enhances temozolomide sensitivity via ROS-amplified ER stress. Its unique mechanism lies in dual redox modulation—simultaneously scavenging ROS and overloading the ER—with nanoparticle formulations under development to improve bioavailability^242^.

Flavonoids further illustrate structural diversity within ER stress modulators^243^,^244^. Quercetin, found in onions and apples, induces SERCA-mediated Ca²⁺ dysregulation and AMPK/mTOR inhibition, thereby promoting autophagy-apoptosis crosstalk in hepatocellular carcinoma and melanoma^245^,^246^. Luteolin, abundant in celery, suppresses WD repeat domain 72 (WDR72)/AKT/EMT signaling in NSCLC and bladder cancers, induces G2/M cell cycle arrest through CHOP upregulation, and synergizes with doxorubicin in resistant lines, highlighting its potential to circumvent drug resistance via ER stress modulation^242^.

Epigallocatechin gallate (EGCG), a green tea catechin, binds to the ATP-binding site of GRP78^247^, thereby disrupting chaperone function and sensitizing breast and glioma cells to etoposide or paclitaxel through JNK/ER stress amplification^248^. Its unconventional aspect is the ability to overcome NF-κB-mediated resistance, with ongoing clinical trials investigating EGCG-curcumin combinations^249^.

Adamantyl-indole derivatives represent a novel synthetic class of Nur77 (Nuclear receptor subfamily 4 group A member 1, NR4A1)-targeting agents that induce ER stress via receptor translocation and UPR activation. A series of N1-(2-(adamantan-1-yl)-1H-indol-5-yl)-N2-(substituent)-1,2-dicarboxamides, derived from indole-urea scaffolds, bind Nur77 with high affinity, promoting its ER localization and activating the PERK-ATF4 and IRE1 pathways. This results in UPR overload, caspase-4 activation, and apoptosis—mechanistically paralleling berberine's PERK/eIF2α activity, but with added Nur77-Bcl-2/translocon-associated protein subunit γ (TRAPγ) crosstalk for enhanced pro-apoptotic signaling. In HCC and breast cancer models, these compounds exhibit potent cytotoxicity, surpassing many flavonoids and EGCG in comparable systems, while maintaining Nur77-dependent selectivity over non-malignant cells^250^.

### 7.2. Coordination compounds targeting ER stress

Platinum compounds remain among the most extensively investigated metal-based anticancer agents^251^. A family of luminescent Pt(II)-NHC complexes selectively localize to the ER and exhibit moderate phototoxicity. Upon irradiation, these complexes induce ER stress, as confirmed by PERK and eIF2α phosphorylation detected by western blotting. ER stress induction is followed by mitochondrial depolarization, caspase activation, and apoptotic cell death^252^.

Ruthenium(II/III) arene complexes are prominent candidates due to Ru's favorable biocompatibility and ligand exchange properties^253^,^254^. One highly hydrophobic dinuclear Ru(II) complex containing 4,7-diphenyl-1,10-phenanthroline (DPP) ligands displays low-micromolar anticancer activity. Fluorescence microscopy confirms ER localization, while the complex exhibits strong liposomal interactions and environment-sensitive luminescence, markedly enhanced in hydrophobic media^255^.

KP1019 (indazolium trans-[RuCl₄(ind)₂]), a Ru(III) prodrug, undergoes reduction under hypoxic tumor conditions to Ru(II), leading to ER Ca²⁺ depletion and activation of IRE1 and PERK, culminating in CHOP-mediated apoptosis. In colorectal xenografts, KP1019 achieves ~50% tumor regression at 100 mg/kg with minimal nephrotoxicity and has advanced to Phase II clinical evaluation. Its unconventional mechanism relies on hypoxia-activated redox switching, enabling bypass of cisplatin resistance through non-DNA targets^256^,^257^.

RM175 ([Ru(η⁶-bip)(en)Cl]+), a Ru(II) piano-stool complex, induces ER stress via mitochondrial-ER Ca²⁺ crosstalk and GRP78 relocalization, with nanomolar IC₅₀ values in breast and prostate cancer models^258^. In vivo, RM175 suppresses PC-3 xenografts by 70%, with photostimulation further enhancing tumor selectivity^259^.

Novel half-sandwich Ru(II) and Os(II) complexes, [Ru(η⁶-p-cym)Cl(L)]PF₆ (1-4) and [Os(η⁶-p-cym)Cl(L)]PF₆ (5-8), featuring polycyclic aromatic hydrocarbon (PAH)-substituted Schiff base ligands (e.g., naphthyl, anthracenyl, phenanthrenyl, pyrenyl), expand this paradigm by integrating DNA intercalation with dual mitochondrial and ER stress induction. These complexes generate ROS, depolarize mitochondria, and activate ER stress-associated genes (e.g., p21/GADD45A), but without prominently engaging canonical UPR branches such as PERK, IRE1, or ATF6. This distinguishes them from KP1019's hypoxia-activated pathways and emphasizes non-inflammatory apoptosis in cisplatin-resistant NSCLC. Os(II) complexes demonstrate superior potency in 2D and 3D lung cancer spheroids, overcoming platinum resistance through ER-mitochondrial crosstalk similar to RM175, but with improved aqueous stability and reduced systemic toxicity. These findings highlight the potential of Os(II) derivatives for combinatorial strategies with radiotherapy in pulmonary malignancies, advancing half-sandwich designs toward clinical translation^260^.

## 8. Discussion and Prospects

ER stress, as a pivotal cellular regulatory hub, holds profound biological significance in tumorigenesis and cancer progression^2^. This stress response system is deeply implicated in shaping the complex TME through its extensive modulation of diverse biological processes, including cellular metabolism, cell cycle regulation, quiescence, invasion and metastasis, angiogenesis, and apoptosis. The activation of the UPR and its associated signaling pathways demonstrates a dual functionality, either promoting or suppressing tumor progression, which reflects the pleiotropic nature of ER stress in cancer biology. This complexity underscores the necessity for comprehensive mechanistic investigations to delineate ER stress-mediated regulatory networks and their therapeutic potential.

Notwithstanding substantial advancements, critical knowledge gaps persist in the field. First, the crosstalk mechanisms between ER stress and other cellular stress responses, such as oxidative stress and the DNA damage response, remain incompletely characterized. Such inter-pathway communication may dynamically balance cellular survival and apoptosis through the coordinated regulation of stress-responsive signaling networks. Second, the mechanisms of ER stress-mediated tumor-immune interactions require further elucidation, particularly concerning their context-dependent effects on various immune cell subsets within the TME. A systematic investigation of these interactions will establish a robust theoretical foundation for the development of innovative cancer immunotherapies.

Future research should prioritize the systematic elucidation of the molecular mechanisms underlying ER stress-induced metabolic reprogramming and transcriptional regulation, with an emphasis on identifying novel ER stress-responsive genes and characterizing their functional roles in tumor progression. To enhance the translational impact of these findings, a promising therapeutic strategy involves the rational combination of ER stress modulators with immune checkpoint inhibitors^7^. The mechanistic synergy for such a combination is strongly supported by evidence of the UPR's role in orchestrating an immunosuppressive TME. For instance, pharmacologically inhibiting specific UPR pathways, such as IRE1α-XBP1, could reverse tumor-intrinsic immune evasion by restoring the expression of MHC-I and MICA, thereby enhancing tumor cell recognition by CD8+ T cells and NK cells, respectively^214^. Concurrently, alleviating ER stress within the TME could directly reinvigorate antitumor immunity by reversing T-cell exhaustion, restoring metabolic fitness, and bolstering effector functions, thus amplifying the pool of cells responsive to checkpoint blockade^220^-^222^. Furthermore, targeting these pathways could reprogram the immunosuppressive myeloid compartment, restoring the antigen-presenting capacity of dendritic cells and attenuating the suppressive functions of MDSCs and TAMs^230^,^232^. This multi-pronged approach provides a robust rationale for combining ER stress modulation with immunotherapy. In-depth exploration of the role of ER stress in maintaining cancer stem cell properties and regulating tumor dormancy is also of critical importance.

However, the clinical translation of ER stress-targeted therapies is fraught with significant challenges that must be surmounted to ensure both efficacy and safety. A primary concern is the potential for on-target, off-tumor toxicities. The UPR is a fundamental process for physiological homeostasis, particularly in professional secretory tissues such as pancreatic β-cells and antibody-producing plasma cells^261^-^263^. Systemic inhibition of key UPR sensors like PERK or IRE1α could disrupt the function of these non-malignant tissues, leading to metabolic disorders, compromised immunity, or other adverse effects. Therefore, developing tumor-selective targeting strategies is crucial for widening the therapeutic window.

Furthermore, the profound intra- and inter-tumoral heterogeneity presents a formidable obstacle. Different cancer types, and even subclones within a single tumor, may exhibit differential reliance on specific UPR branches for survival^264^. This variability implies that patient response to a given UPR inhibitor will likely depend on the unique molecular signature of their malignancy. Consequently, the development of predictive biomarkers to stratify patients and guide therapeutic selection is imperative for the successful clinical implementation of these agents.

A final major hurdle is the emergence of adaptive resistance mechanisms. Cancer cells often exhibit remarkable plasticity; upon inhibition of one UPR pathway, they can bypass the therapeutic blockade by compensatory up-regulation of another. For instance, prolonged ATF6 inhibition might lead to a compensatory enhancement of IRE1α or PERK signaling, allowing tumor cells to restore proteostasis and survive^265^. This adaptability suggests that combination strategies, involving either simultaneous or sequential targeting of multiple UPR nodes, may be necessary to preempt the evolution of resistant clones and achieve durable clinical responses.

In conclusion, while ER stress represents a highly promising and multifaceted therapeutic target, its central role in both normal physiology and cancer biology introduces complex clinical challenges. Overcoming issues of toxicity, tumor heterogeneity, and adaptive resistance will be paramount. Through comprehensive characterization of ER stress signaling networks and the development of precision-targeted intervention strategies, particularly those designed to synergize with existing treatments, the translational application of these fundamental research findings may ultimately improve clinical outcomes for cancer patients.

## Abbreviations

ACL: ATP citrate lyase
ACLY: ATP citrate lyase
AKT1: protein Kinase B
AMPK: AMP-activated protein kinase
ASK1: apoptosis signal-regulating kinase 1
ASNS: asparagine synthetase
ATF6: activating transcription factor 6
ATG5: autophagy-related protein
BCL-2: B-cell lymphoma-2
BIM: Bcl-2 interacting mediator of cell death
BMP2: bone morphogenetic protein 2
BRAF: B-Raf proto-oncogene
BZW1: basic leucine zipper and W2 domains 1
C/EBP: CCAAT/enhancer-binding protein
CAF: cancer-associated fibroblast
CCPG1: cell-cycle progression gene 1
CDP: cytidine diphosphate
CHOP: C/EBP homologous protein
CLL: chronic lymphocytic leukemia
CLPTM1L: calcium phosphate binding protein 1-like
CRE: cyclic AMP response element
CREB: cAMP response element-binding protein
CRR9: cisplatin resistance-related protein 9
DC: dendritic cell
DDRGK: DDRGK domain containing 1
DPP: 4,7-diphenyl-1,10-phenanthroline
DTC: disseminated tumor cell
EDEM: ER degradation-enhancing alpha-mannosidase-like protein
EGCG: epigallocatechin gallate
eIF2α: eukaryotic translation initiation factor 2 subunit alpha
EMT: epithelial-mesenchymal transition
ER: endoplasmic reticulum
ERAD: ER-associated degradation
ERdj4: ER-localized DnaJ homolog 4
ERO-1α: ER oxidoreductase 1 alpha
ERO1α: ER oxidoreductin 1 alpha
ERP44: ER protein 44
ERSE: ER stress response element
FADD: Fas-associated protein with death domain
FAS: fatty acid synthase
FASN: fatty acid synthase
FATP: fatty acid transport protein
FGF2: fibroblast growth factor 2
Foxp3: forkhead box P3
GABARAP: gamma-aminobutyric acid receptor-associated protein
GADD34: growth arrest and DNA damage-inducible 34
GCN2: general control nonderepressible 2
GLS1: glutaminase 1
GRP78: glucose-Regulated Protein 78
GSH: glutathione
HCC: hepatocellular carcinoma
HDAC1: histone deacetylase 1
HEDJ: Hsp40 ER-associated DnaJ homolog
HIF-1α: hypoxia-inducible factor-1α
HMGA2: high mobility group AT-hook 2
HRAS: Harvey rat sarcoma virus gene
IFN-γ: interferon-gamma
IL-6: interleukin-6
INS1: insulin 1
IP3R: inositol trisphosphate receptor
IRE1: inositol-requiring enzyme 1
IRES: internal ribosome entry site
ISR: integrated stress response
ITPR3: inositol 1,4,5-trisphosphate receptor type 3
JNK: c-Jun N-terminal kinase
KEAP1: ECH-associated protein 1
LDHA: lactate dehydrogenase A
MAM: mitochondria-associated ER membrane
MAPK: mitogen-activated protein kinase
MDSC: myeloid-derived suppressor cell
MFN2: mitofusin 2
MHC-I: major histocompatibility complex class I
MICA: MHC class I polypeptide-related sequence A
mtDNA: mitochondrial DNA
mTOR: mechanistic target of rapamycin
mTORC1: mTOR complex 1
NADPH: nicotinamide adenine dinucleotide phosphate-oxidase
NBR1: neighbor of BRCA1 gene 1
NF-kB: nuclear factor-kappa B
NK: natural killer
NKG2D: natural killer group 2D
NLRP3: NOD-like receptor protein 3
NR4A1: nuclear receptor subfamily 4 group A member 1
NRF2: nuclear factor erythroid 2-related factor 2
NSCLC: non-small cell lung cancer
OASIS: old astrocyte specifically induced substance
p58IPK: 58 kDa inhibitor of protein kinase
PC1: prohormone convertase 1
PDAC: pancreatic ductal adenocarcinoma
PDGF: platelet-derived growth factor
PDI: protein disulfide isomerase
PD-L1: programmed death-ligand 1
PERK: protein kinase RNA-activated
PGE2: prostaglandin E2
PINK1: PTEN-induced putative kinase 1
PKM2: pyruvate kinase M2
PLCγ: phospholipase Cγ
PMN: polymorphonuclear neutrophils
PP2A: protein phosphatase 2A
PPARγ: peroxisome proliferator-activated receptor gamma
PRL11: prolactin receptor-like protein 11
PTEN: Phosphatase and tensin homolog
RACK1: receptor for activated c kinase 1
RAMP-4: ribosome-associated membrane protein 4
RIDD: regulated IRE1-dependent RNA decay
RIG1: retinoic acid-inducible gene
ROS: reactive oxygen species
SACC: salivary adenoid cystic carcinoma
SCD1: stearoyl-CoA desaturase 1
SERCA: sarco/endoplasmic reticulum calcium ATPase
sIgM: secretory immunoglobulin M
SLC1A5: solute carrier family 1 member 5
SREBP: sterol regulatory element-binding protein
STAT3: signal transducer and activator of transcription 3
STING: stimulator of interferon genes
SYP: synaptophysin
TAD: transcriptional activation domain
TAM: tumor-associated macrophages
TAP1: transporter associated with antigen processing 1
TCA: tricarboxylic acid
TG: thapsigargin
TGF-β: transforming growth factor-beta
TME: tumor microenvironment
TNBC: triple-negative breast cancer
TRAF2: TNF receptor-associated factor 2
TRAIL: TNF-related apoptosis-inducing ligand
TRAPγ: translocon-associated protein subunit γ
Treg: regulates regulatory T cell
TRIM25: tripartite motif-containing 25
TXNIP: thioredoxin interacting protein
UDP-GlcNAc: uridine diphosphate-N-acetylglucosamine
uORF: upstream open reading frame
UPR: unfolded protein response
VCP: valosin-containing protein
VEGF: vascular endothelial growth factor
VLDL: very low-density lipoproteins
WDR72: WD repeat domain 72
XBP1: X-box-binding protein 1
